# Modulation of HIV-1 infectivity and cyclophilin A-dependence by Gag sequence and target cell type

**DOI:** 10.1186/1742-4690-6-21

**Published:** 2009-03-02

**Authors:** Saori Matsuoka, Elisabeth Dam, Denise Lecossier, François Clavel, Allan J Hance

**Affiliations:** 1 INSERM U941, Paris 75010, France; 2 Institut Universitaire d'Hématologie, Université Paris Diderot, Paris 75010, France; 3 BioAlliancePharma, Paris 75015, France

## Abstract

**Background:**

HIV-1 Gag proteins are essential for virion assembly and viral replication in newly infected cells. Gag proteins are also strong determinants of viral infectivity; immune escape mutations in the Gag capsid (CA) protein can markedly reduce viral fitness, and interactions of CA with host proteins such as cyclophilin A (CypA) and TRIM5α can have important effects on viral infectivity. Little information, however, is available concerning the extent that different primary Gag proteins affect HIV-1 replication in different cell types, or the impact on viral replication of differences in the expression by target cells of proteins that interact with CA. To address these questions, we compared the infectivity of recombinant HIV-1 viruses expressing Gag-protease sequences from primary isolates in different target cells in the presence or absence of agents that disrupt cyclophilin A – CA interactions and correlated these results with the viral genotype and the expression of cyclophilin A and TRIM5α by the target cells.

**Results:**

Viral infectivity was governed by the nature of the Gag proteins in a target cell-specific fashion. The treatment of target cells with agents that disrupt CypA-CA interactions often produced biphasic dose-response curves in which viral infectivity first increased and subsequently decreased as a function of the dose used. The extent that treatment of target cells with high-dose CypA inhibitors impaired viral infectivity was dependent on several factors, including the viral genotype, the nature of the target cell, and the extent that treatment with low-dose CypA inhibitors increased viral infectivity. Neither the presence of polymorphisms in the CA CypA-binding loop, the level of expression of CypA, or the level of TRIM5α expression could, alone, explain the differences in the shape of the dose-response curves observed or the extent that high-dose CypA inhibitors reduced viral infectivity.

**Conclusion:**

Multiple interactions between host-cell factors and Gag can strongly affect HIV-1 infectivity, and these vary according to target cell type and the origin of the Gag sequence. Two of the cellular activities involved appear to be modulated in opposite directions by CypA-CA interactions, and Gag sequences determine the intrinsic sensitivity of a given virus to each of these cellular activities.

## Background

The HIV-1 Gag proteins play important roles throughout the viral life-cycle, including the assembly and release of viral particles, their subsequent maturation into infectious virions, and during the events occurring between the release of capsids into newly infected cells and the integration of proviral DNA. During the early steps of the viral life cycle, viral proteins, especially capsid (CA), are in intimate contact with the intracellular environment. Considerable evidence supports the idea that interactions between host cellular proteins and the viral capsid are important for events occurring early in infection, such as the transport of the preintegration complex, uncoating of the capsid, nuclear entry, and integration (reviewed in [1-4]).

A striking example of such interactions is that occurring between the capsid and the abundant intracellular protein cyclophilin A (CypA), a peptidyl-prolyl isomerase whose active site binds a proline residue present in an exposed loop extending from the CA subunits [5,6]. Several lines of evidence indicate that the inhibition of CypA-CA interactions in newly infected human target cells usually impairs viral infectivity, including studies evaluating the infection of target cells whose CypA expression has been reduced or eliminated, the effect of inhibiting CypA-CA interactions using cyclosporine A (CsA) or its analogs, and the impact on infectivity of CA mutations such as P90A and G89A that impair CypA binding [5,7-15]. Although inhibition of CypA-CA interactions has generally been found to be deleterious to HIV-1 replication in human cells, exceptions have been reported. Viruses carrying CA mutations selected during viral replication in CsA-treated target cells (A92E, G94D) and a mutation produced through alanine scanning (T54A) replicate better in some, but not all, target cells in the presence of CsA [10,11,16-18]. Because these mutants continue to bind CypA, the results indicate that CypA binding can also be detrimental to HIV-1 replication in a virus-specific and target cell-specific fashion. The mechanisms through which CypA binding modulates viral infectivity are not defined and several possibilities have been discussed, including effects on capsid stability, viral uncoating, and the protection of viral cores from cellular restriction factors [8,19-23].

The HIV-1 CA is also known to be targeted by host cell restriction factors, including the well characterized TRIM5α protein and the activity designated as Lv2 [7,9,24-33]. Although human TRIM5α can inhibit the replication of a variety of retroviruses to various extents (N-MLV, EIAV, HIV-2, FIV, SIVmac), it displays only modest activity against HIV-1 [7,34-41]. Interestingly, human TRIM5α is more active against HIV-1 expressing the G89V mutation than against wild-type HIV-1 [39], but less active against viruses carrying certain polymorphisms in the CypA binding loop [21,24,27,30,42-44], consistent with the possibility that CypA binding may modulate the activity of human TRIM5α.

The viral strain-dependent effects of CypA and TRIM5α interactions described above underscore the potential importance of Gag polymorphisms on HIV-1 replication capacity. In particular, it has been well documented that a number of Gag mutations selected in response to immune pressure can be deleterious to viral replication [45-47]. It remains unclear, however, whether these polymorphisms modify intrinsic properties of the capsid structure or influence the ability of Gag proteins to interact with host cellular proteins. Because the expression of cellular proteins that can interact with the viral capsid is likely to differ in different cell types, the finding that the replicative capacity of a virus expressing a given *gag *gene is cell-type dependent would support the later possibility. Furthermore, little information is available on the replication of viruses expressing Gag proteins derived from primary HIV-1 isolates in different cell types, and the replicative impact of the level of expression of cellular proteins that interact with viral capsids from such viruses is unknown.

To address these questions, we compared the infectivity of recombinant HIV-1 viruses expressing Gag-protease (Gag-PR) sequences from primary isolates in different target cells in the presence or absence of agents that disrupt CypA-CA interactions and correlated these results with the viral genotype and the expression of CypA and TRIM5α by the target cells. Our results indicate that viral infectivity is governed by the nature of the Gag proteins in a target cell-specific fashion. The treatment of target cells with agents that disrupt CypA-CA interactions often produce biphasic dose-response curves in which viral infectivity first increased, and subsequently decreased as a function of the dose used. The extent that treatment of target cells with high-dose CypA inhibitors impairs viral infectivity is dependent on several factors, including the viral genotype, the nature of the target cell, and the extent that treatment with low-dose CypA inhibitors increased viral infectivity. Neither the presence of polymorphisms in the CA CypA-binding loop, the level of expression of CypA, or the level of TRIM5α expression could, alone, explain the differences in the shape of the dose-response curves observed or the extent that high-dose CypA inhibitors reduced viral infectivity. We conclude from these observations that the impact of CypA antagonists on viral infectivity is multi-factorial and may reflect both the relative abundance and the viral susceptibility to two cellular activities, whose effect on HIV-1 infectivity is modulated in opposite directions by CypA-CA interactions.

## Results

### Infectivity of recombinant viruses in different target cells

We created a series of pNL4-3 based recombinant viruses in which the Gag-PR sequences were derived from clinical isolates obtained from patients who had never received protease inhibitors, and in which a sequence coding *Renilla *luciferase was inserted in place of Nef. In each case, proviruses expressing the NL4-3 (X4-tropic) envelope as well as envelope-deleted versions were produced. In initial studies, VSV-pseudotyped envelope-deleted viruses were used to infect different target cells, and 40 hours later, infectivity was assessed by measuring luciferase expression.

**Figure 1:**
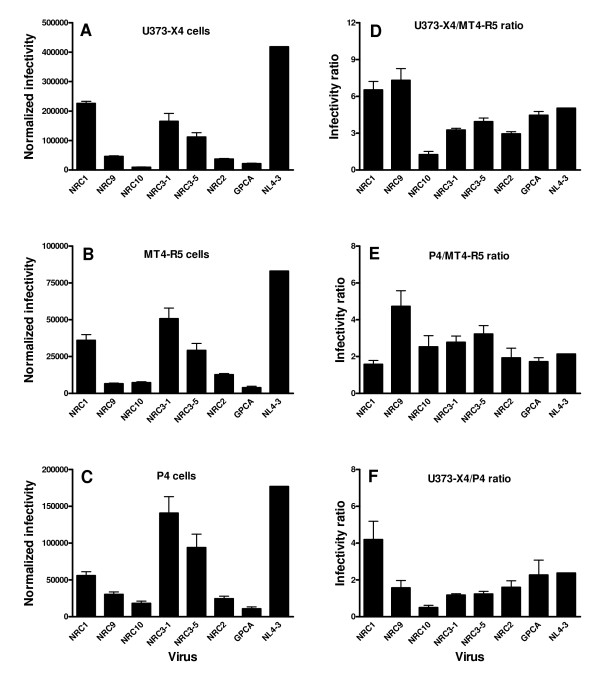
**Infectivity of recombinant viruses in different cell types**. (A-C) The indicated target cells were cultured overnight in 96-well plates, infected with serial dilutions of supernatants containing each of the 7 indicated VSV pseudotyped envelope-deleted recombinant viruses (final concentrations 0.156 – 10 ng p24/ml), and luciferase activity (RLU) expressed by the target cells was determined 40 h after infection. The slope of the dose response curve was determined by linear regression. The slope obtained from infection of target cells by NL4-3 was measured on each 96-well plate, and the value used to normalize the slope of the other samples present on that plate, as described in the Materials and Methods. The results shown are the mean ± SEM for 4 independent experiments. (D-E) For each experiment, the results were also expressed as a ratio of the normalized slopes obtained for the two indicated target cell types. The results shown are the mean ± SEM for 4 independent experiments.

The amount of luciferase activity produced was cell-type dependent. Thus, for a given virus, greater luciferase activity was always observed using U373-X4 cells than in MT4-R5 cells or P4 cells (compare Figures 1A–C). In addition, some viruses appeared to have generally good (NL4-3) or poor infectivity (NRC10). Nevertheless, by comparing the ratio of luciferase activity in two different cell types it was clear that the relative infectivity of these viruses for different cell types could be quite different. For example, the ratio of luciferase activities observed in U373-X4 cells/MT4-R5 cells was elevated and not significantly different for the viruses NRC1 and NRC9 (Figure 1D), but the ratio of luciferase activities observed in P4 cells/MT4-R5 cells was significantly lower for NRC1 than for NRC9 (p < 0.001, Figure 1E), whereas the ratio of luciferase activities observed in U373-X4 cells/P4 cells was significantly greater for NRC1 than NRC9 (p < 0.001, Figure 1F). Thus, the Gag-Pol sequences expressed by NRC1 promoted replication in U373-X4 cells relative to that in P4 cells, whereas the converse was true for NRC9.

Similarly, the U373-X4/MT4-R5 and U373-X4/P4 ratios were greater for NRC2 than for NRC10 (p < 0.05 for both comparisons). In contrast, the P4/MT4-R5 ratios for NRC2 and NRC10 were not significantly different, consistent with the idea that the gag-pol sequences expressed by NRC2 were more favorable to replication in U373-X4 cells than those expressed by NRC10. Very similar results were obtained when recombinant viruses expressing the NL4-3 envelope were used in the place of VSV-pseudotyped viruses (data not shown), suggesting that the entry pathway did not have a major impact on these cell-type specific differences in infectivity.

To evaluate the impact of mutations that impair protease activity on the relative infectivity of viruses in different cell types, we also evaluated a recombinant virus (GPCA) that expresses the same Gag sequence as NL4-3, but contains 3 resistance mutations in the protease that reduce infectivity to 20% of that of NL4-3 (Figures 1A–C). The relative infectivity of GPCA in different cell types was not significantly different from that of NL4-3 (Figures 1D–F).

Taken together, these findings indicate that: i) target cells differentially express activities that modulate viral infectivity, and that ii) the impact of these activities on infectivity varies as a function of the Gag-PR sequences expressed by the viruses.

### Effect of cyclophilin inhibitors on viral infectivity in different target cells

**Figure 2:**
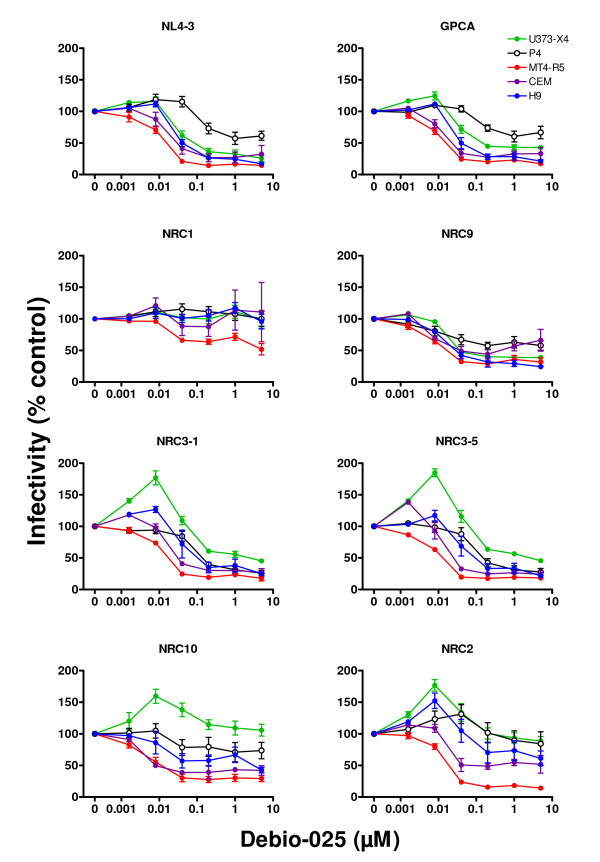
**Effect of Debio-025 on the infectivity of recombinant viruses**. Target cells, [U373-X4 (filled green circles), P4 cells (open black circles), MTR-R5 cells (filled red circles), CEM cells (filled purple circles), and H9 cells (filled blue circles)] were cultured in the presence of serial dilutions of Debio-025 and infected with 100 ng p24/ml (all recombinant viruses except NL4-3) or 20 ng p24/ml (NL4-3) of the indicated recombinant viruses. Luciferase activity (RLU) expressed by target cells was determined 40 h after infection. The results are expressed as a percentage of luciferase activity in cells cultured in the absence of Debio-025. As indicated in the Materials and Methods, several dilution schemes were used in the course of these studies; each data point represents pooled data for cells incubated the presence of a range of concentrations of Debio-025 as follows: 5 μM (2–5 μM), 1 μM (0.5–1 μM); 200 nM (125–200 nM); 40 nM (30–40 nM); 8 nM (8–10 nM); 1.6 nM (1.6 nM). The results shown are the mean ± SEM for 6 independent experiments (U373-X4, P4, MT4-R5) or 3 independent experiments (CEM, H9).

Previous studies have indicated that interactions between viral capsid and cyclophilin A can modulate viral infectivity. To further characterize the activities expressed by different target cells that influence viral infectivity, we evaluated the impact of the disruption of capsid-CypA interactions on the infectivity of the 7 different recombinant viruses in 5 different target cell types. The results obtained using the CsA analog Debio-025 are shown in Figure 2. A variety of response profiles were observed, depending on both which recombinant virus was evaluated and the nature of the target cell. When MT4-R5 cells were used as target cells, treatment with increasing doses between 0.16 nM and 40 nM Debio-025 led to a progressive decrease in infectivity. At higher doses infectivity often reached an apparent plateau level that could differ for different viruses (e.g., the infectivities of NRC10 and NRC2 in cells treated with 5 μM Debio-025 were, respectively, 29.3 ± 12.3% and 14.0 ± 6.8% of that observed in untreated cells, p < 0.03 by t-test).

When U373-X4 cells were used as target cells, the infectivity of some viruses (NRC3-1, NRC3-5, NRC2, NRC10) increased by more than 50% following treatment with 1.6 – 8 nM Debio-025 (p < 0.04 – 0.01). In U373-X4 cells treated with higher doses of Debio-025 (40 nM – 5 μM), the infectivity of all viruses except NRC1 decreased significantly compared to that observed in cells treated with 8 nM Debio-025. At the highest doses of Debio-025 tested (5 μM), the percent residual infectivity of each virus in U373-X4 cells was always significantly higher than that observed with similarly treated MT4-R5 cells, but varied from 25.6 ± 12.0% (NL4-3) to 105.8 ± 22.5% (NRC10) of that observed in untreated U373-X4 cells.

When HeLa-derived P4 cells, CEM cells and H9 cells were used as targets, the profiles of infectivity generally fell in between those seen for MT4-R5 cells and U373-X4 cells. Following treatment with low doses of Debio-025, significant increases in infectivity were observed using CEM and H9 cells for several viruses, but the magnitude of this effect was less striking than that seen in U373-X4 cells. At higher doses, infectivity in CEM, H9 and P4 cells decreased for all viruses except NRC1. The extent of inhibition observed in these cell types following pretreatment with 5 μM Debio-025, however, was often less than that observed in similarly treated MT4-R5 cells. For most viruses, the inhibition of infectivity in CEM, H9 and P4 cells pretreated with 5 μM Debio-025 was greater than that observed in similarly treated U373-X4 cells, but exceptions were observed (e.g., NL4-3 and NRC9 in P4 cells, NRC9 in CEM cells).

**Figure 3:**
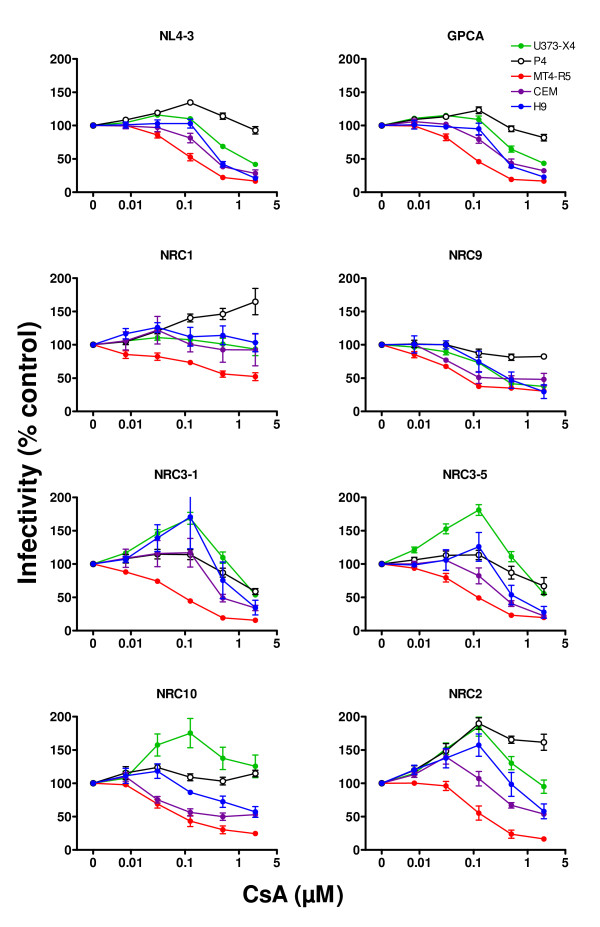
**Effect of cyclosporine A on the infectivity of recombinant viruses**. Target cells, [U373-X4 (filled green circles), P4 cells (open black circles), MTR-R5 cells (filled red circles), CEM cells (filled purple circles), and H9 cells (filled blue circles)] were cultured in the presence of the indicated serial dilutions of CsA and infected with 100 ng p24/ml (all recombinant viruses except NL4-3) or 20 ng p24/ml (NL4-3) of the indicated recombinant viruses. Luciferase activity (RLU) expressed by target cells was determined 40 h after infection. The results are expressed as a percentage of luciferase activity in cells cultured in the absence of CsA. The results shown are the mean ± SEM for 6 independent experiments (U373-X4, P4, MT4-R5) or 3 independent experiments (CEM, H9).

Similar studies were performed using using the CypA inhibitor CsA. As shown in Figure 3, the profiles were generally similar to those observed using Debio-025, except that the curves were shifted to the right. When U373-X4 cells were used as target cells, the infectivity of the same 4 viruses (NRC3-1, NRC3-5, NRC2, NRC10) increased by more than 50% following treatment with 100 nM CsA. The dose of CsA required for maximal increase in the infectivity of these viruses, however, was more than 10-fold higher than that required for Debio-025 (8 nM). For each virus, the extent of inhibition of viral infectivity observed in U373-X4 cells and MT4-R5 cells treated with 2 μM CsA was generally similar to that seen when these cells were treated with 5 μM Debio-025. Consistent with the lower potency of CsA, evidence of an inhibitory plateau frequently observed in Debio-025 treated cells was usually less evident for CsA-treated cells.

The Debio-025 and CsA dose-response curves for GPCA, the virus expressing the same gag sequence as NL4-3 but containing protease mutations that impair infectivity, were very similar to those obtained for NL4-3.

### Relationship between the effect of low-dose and high-dose Debio-025 on viral infectivity

**Figure 4:**
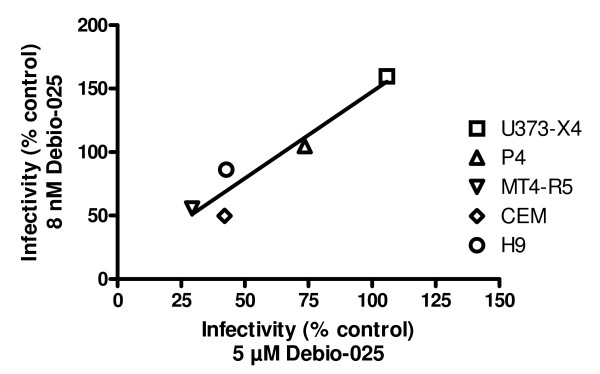
**Relationship between the viral infectivity in cells treated with low-dose and high-dose Debio-025**. The infectivity of the recombinant virus NRC-10 in cells treated with 8 nM Debio-025 (low-dose Debio-025) is plotted as a function of the infectivity in cells treated with 5 μM Debio-025 (high-dose Debio-025) for each of the indicated target cells. The linear regression for these results is also shown (r^2 ^= 0.91, p < 0.02).

For several viruses, as shown above, the effect of treatment of certain target cells with Debio-025 on infectivity gave biphasic dose-response curves in which viral infectivity first increased and then subsequently decreased as a function of the dose of Debio-025 used. To examine the possibility that the increase in infectivity observed in cells treated with low-dose Debio-025 might influence the extent of inhibition observed when cells were treated with high-dose Debio-025, regression analyses were performed for each virus. As illustrated for NRC10 in Figure 4, a direct correlation was observed comparing the infectivity observed at 8 nM Debio-025 and that observed with 5 μM Debio-025 (r^2 ^= 0.91, p < 0.02). Overall, for the 4 viruses displaying a more than 50% increase in infectivity in at least one target cell type treated with 8 nM Debio-025 (NRC3-1, NRC3-5, NRC10, NRC2), significant positive correlations were observed in 3 cases (r^2 ^0.86 – 0.92; p < 0.01 – 0.03), and for the fourth virus (NRC2), a relatively strong correlation was also seen although it did not achieve statistical significance (r^2 ^= 0.66, p = 0.09). It is also noteworthy that among the target cells tested, MT4-R5 cells were the only target cells in which a significant increase in infectivity was not observed for any of the viruses following treatment with 8 nM Debio-025. Indeed, for all viruses except NRC1, treatment of MT4-R5 cells with 8 nM Debio-025 led to a significant decrease in viral infectivity, ranging from 18.4% for NRC2 to 45.2% for NRC10.

### Cyclophilin A expression in target cells

**Figure 5:**
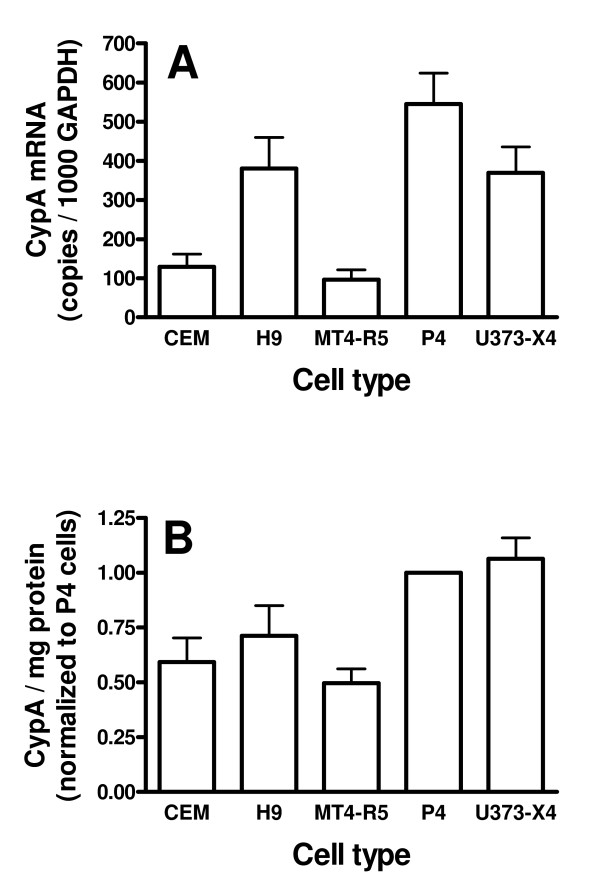
**CypA expression in target cells**. (A) CypA mRNA levels, expressed as copies per 1000 copies of GAPDH mRNA, were determined by real-time PCR using primers and probes shown in Table 2. Results are the mean ± SEM for 3 independent experiments using different cell pellets. (B) CypA protein expression. Equal amounts of soluble cell protein were electrophoresed into 10% SDS-PAGE gels, and transferred to PVDF membranes. The blots were probed sequentially with a rabbit anti-cypA antibody and a IRDye 800CW-conjugated donkey anti-rabbit IgG antibody. The blots were scanned using an Odyssey Infrared imaging system, and fluorescence intensity was analysed using Odyssey application software. Lysates from P4 cells were included in each gel, and the intensities of the CypA bands in the other samples on the same gel were expressed relative to that observed for P4 cells. The results presented are the mean ± SEM for 4 independent experiments, all performed using different cell suspensions.

Previous studies have suggested that the level of expression of CypA in target cells may influence the effect of CsA or CsA analogs on viral infectivity [11,21,48-50]. To explore this relationship for the target cells and viruses evaluated in this study, we measured CypA expression at both the mRNA and protein level. CypA mRNA expression relative to the housekeeping gene glyceraldehyde-3-phosphate dehydrogenase (GAPDH) varied over a 5.6-fold range (Figure 5A). At the mRNA level, the expression of CypA was significantly higher in both P4 and U373-X4 cells than in CEM cells or MT4-R5 cells (p < 0.05 – 0.01 for all comparisons). At the protein level, CypA expression varied over only a 2-fold range (Figure 5B). CypA expression per milligram of protein was significantly higher in both P4 and U373-X4 cells than in CEM cells, MT4-R5 cells or H9 cells (p < 0.01 for all comparisons).

The comparison of the results of CypA expression (Figure 5) and the dose-response curves for Debio-025 and CsA (Figures 2 and 3, respectively) did not support the conclusion that differences in cellular CypA levels could explain the occurrence of biphasic responses in some, but not all, cell types. Despite the fact that, CypA expression in U373-X4 and P4 cells was not significantly different at either the mRNA or protein level, treatment of U373-X4 cells with 8 nM Debio-025 led to a greater than 50% increase in infectivity for 4/8 viruses tested. In contrast, increases of this magnitude were never seen in P4 cells. Conversely, following pretreatment with 8 nM Debio-025, significant increases in infectivity were observed for some viruses in H9 and/or CEM cells, despite the fact that CypA expression was lower in these cell types than in P4 cells.

CypA expression also did not appear to explain the extent that treatment of target cells with high-dose Debio-025 reduced viral infectivity. As noted above, treatment of cells with doses greater than 200 nM Debio-025 resulted in an apparent plateau effect, suggesting that CA-CypA interactions had been completely inhibited in all cell types at doses above 100 nM. Nevertheless, the extent of inhibition of infectivity of several viruses (e.g. NRC9, NRC3-5) could be similar in cell types expressing different levels of CypA, and inhibition by high-dose Debio-025 was often greatest in MT4-R5 cells, the cell line that expressed the lowest levels of CypA.

### TRIM5α expression in target cells

**Figure 6:**
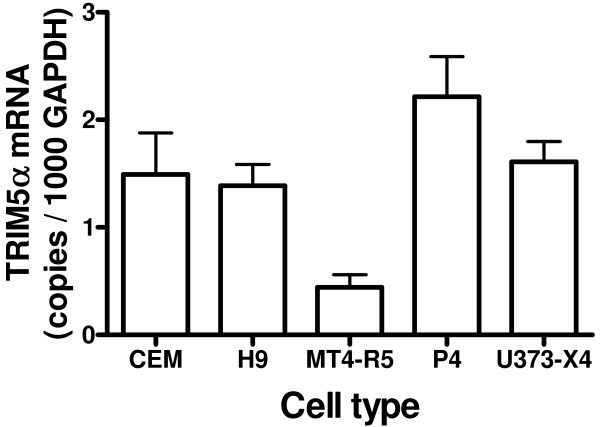
**TRIM5α expression in target cells**. TRIM5α mRNA levels, expressed as copies per 1000 copies of GAPDH mRNA, were determined by real-time PCR using primers and probes shown in Table 2. Results are the mean ± SEM for 3 independent experiments using different cell pellets.

We also evaluated the expression of the restriction factor TRIM5α at the mRNA level. TRIM5α mRNA expression relative to GAPDH varied over a 5-fold range (Figure 6). The TRIM5α/GAPDH ratio was significantly lower in MT4-R5 cells than in the other four target cells evaluated (p < 0.01 – 0.05). CypA mRNA levels were considerably higher than TRIM5α mRNA levels in all cell types, ranging from 86-fold (CEM cells) to 275-fold (H9 cells).

Because TRIM5α expression was similar in all target cell types except MT4-R5 cells, these findings do not support the idea that differences in TRIM5α expression explain the occurrence of biphasic dose-response curves in some, but not all, target cells. The greatest inhibition by high-dose Debio-025 usually occurred in MT4-R5 cells, the cell line that had the lowest TRIM5α/GAPDH ratio.

### Genotype-phenotype correlations

The amino acid sequences of the CA proteins surrounding the CypA binding loop for the 7 viruses are shown in Table 1. The infectivity of NRC1 was not modified after treatment of most target cells with Debio-025, although infectivity did decrease significantly in MT4-R5 cells. NRC1 expressed mutations in and near the CypA binding loop (V86A/H87Q/I91V/M96L) at positions that have been shown to confer CypA resistance in several previous studies [21,24,27,30,42-44]. NRC9 also expressed two of these mutations. The infectivity of this virus was inhibited in Debio-025 treated cells, but the extent of inhibition of this virus in Debio-025 treated MT4-R5 cells was less than that observed for the other viruses studied. Viruses demonstrating more that a 50% increase in infectivity in target cells treated with 8 nM Debio-025 had two (NRC10), one (NRC2) or no mutations (NRC3-1, NRC3-5) in the CypA binding loop, although it is perhaps noteworthy that the later two viruses did have the L83V mutation in a residue adjacent to this region.

**Table 1:** Sequences of the region surrounding the CypA binding Loop in CA*

**Virus**	**Position and Sequence**																				
--- CypA Binding Loop ---																					
	80	81	82	83	84	*85*	*86*	*87*	*88*	*89*	*90*	*91*	*92*	*93*	94	95	96	97	98	99	100
**NL4-3**	W	D	R	L	H	P	V	H	A	G	P	I	A	P	G	Q	M	R	E	P	R
**NRC1**	.	.	.	.	.	.	A	Q	.	.	.	V	.	.	.	.	L	.	.	.	.
**NRC2**	.	.	.	.	.	.	.	.	.	.	.	V	.	.	.	.	.	.	.	.	.
**NRC3-1**	.	.	.	V	.	.	.	.	.	.	.	.	.	.	.	.	.	.	.	.	.
**NRC3-5**	.	.	.	V	.	.	.	.	.	.	.	.	.	.	.	.	.	.	.	.	.
**NRC9**	.	.	.	.	.	.	A	.	.	.	.	V	.	.	.	.	.	.	.	.	.
**NRC10**	.	.	.	.	.	.	A	.	.	.	.	.	.	.	.	.	I	.	.	.	.

*Numbers indicate the amino acid position in the CA protein. The CypA binding loop is indicated in italics [19]. The amino acid sequence for NL4-3 is given in single letter code. Only amino acids that differ from the NL4-3 sequence are shown for the other viruses.

Mutations outside of the CypA binding loop have been shown to modify the sensitivity of viruses to CypA inhibitors, including T54A, A105T, T110N, N121S and R132K [18,27,45,46]. None of the viruses studied here carried mutations at positions 54, 105 or 121. NRC1 expressed the T110N mutation, a T-cell epitope escape mutation in HLA-B57+ individuals that can reduce infectivity and increase sensitivity to CsA. As indicated above, however, this virus also expressed compensatory mutations in the CypA binding loop that restore infectivity and impart CsA resistance to viruses carrying the T110N mutation [45]. One virus (NRC10) expressed the R132K and L136M mutations, T-cell epitope escape mutations in HLA-B27+ individuals that cause an impairment in viral replication in CEM cells that can be partially restored by culturing target cells with 0.5 μM CsA [46]. In our studies, NRC10 did show poor infectivity in all target cells tested. Although viral infectivity was inhibited by treatment of most cell types with high-dose CsA and Debio-025, it was one of four viruses that demonstrated biphasic dose response curves, consistent with the possibility that the R132K mutation contributed to this phenotype for this virus.

## Discussion

This study, comparing the infectivity of recombinant HIV-1 viruses expressing Gag-PR sequences from primary isolates in different target cells in the presence or absence of agents that disrupt CypA-CA interactions, established several interesting points: i) viral infectivity is governed both by the nature of the Gag proteins expressed and the nature of the target cells; ii) the treatment of target cells with Debio-025 or CsA often produces biphasic dose-response curves; iii) the extent that treatment of target cells with high-dose CypA inhibitors impairs viral infectivity is dependent on several factors, including the virus used, the nature of the target cell, and the extent that treatment with low-dose CypA inhibitors increased viral infectivity; and iv) neither the level of expression of CypA nor the level of TRIM5α could, alone, explain the differences in the shape of the dose-response curves observed or the extent that high-dose CypA inhibitors reduced viral infectivity. Each of these observations is discussed below.

The relative infectivity of the recombinant HIV-1 viruses evaluated here in different cell types and in different conditions was clearly influenced by the primary Gag-PR sequence expressed by the virus. In these studies, we chose to use recombinant viruses expressing both Gag and protease sequences from the primary isolate to ensure that all Gag proteins expressed by the recombinant viruses were derived from the primary isolate (including the C-terminal region of p6), and to ensure optimal compatibility between the protease and its Gag substrate. Although polymorphisms in the protease could also modify viral infectivity, it is most likely that the cell type-dependent differences in infectivity seen in our experiments reflect differences in Gag proteins. First, the primary isolates were obtained from patients with no exposure to protease inhibitors. Second, a NL4-3 derived virus carrying 3 mutations in protease (L10I, G48V, V82A) that reduced viral infectivity by 5-fold had no significant impact on either the relative infectivity in different cell types, or on the profiles of the dose-response curves obtained using CypA inhibitors compared to those observed for wild-type NL4-3. These results support the conclusion that the target cells used in this study differentially express factors whose ability to modify viral infectivity is dependent on the structure of the Gag proteins expressed by the virus, not differences in protease activity.

A striking finding in our study was the observation that the treatment of target cells with CsA or Debio-025 often produced biphasic dose-response curves in which infectivity increased following treatment with low doses of the CypA inhibitor, but decreased following treatment with higher doses. The presence and extent of such biphasic responses were dependent, however, on both the nature of the virus and the target cell employed. Studies evaluating the full dose-response curve to CypA inhibitors using infectivity assays have not been previously reported. Using other approaches, treatment of target cells with CsA has been found to increase viral replication in a dose-dependent and cell-type dependent fashion. For example, in studies evaluating virus accumulation after multiple replicative cycles, both Gatanaga et al. and Yin et al. [21,49] found that the replication of NL4-3 in H9 cells was improved by treatment with 0.5 μM CsA but not 2.5 μM CsA, whereas both doses inhibited NL4-3 replication in Jurkat cells and mitogen-stimulated lymphocytes. In our experiments, both of these doses of CsA inhibited NL4-3 infectivity in H9 cells. Because multi-cycle viral replication assays are sensitive not only to effects of CsA on the early steps of viral replication, as measured in our experiments, but also to potential effects on viral production [51,52] and the infectivity of the viruses released [10,11,14,53,54], it is not surprising that the two approaches would not give completely concordant results. With one possible exception, Ptak et al. [55] did not observe biphasic dose response curves when the replication of 18 different viral isolates was evaluated in Debio-025 treated mitogen-stimulated lymphocytes.

The increase in viral infectivity observed following treatment of certain target cells with low-dose CypA inhibitors is also somewhat reminiscent of responses observed for HIV-1 carrying the A92E or G94D mutations in CA, mutations that were selected after the in vitro replication of wild-type virus in CsA-treated target cells [16]. Depending on the target cell type used, replication of these mutants is either improved (CsA dependent phenotype) or unmodified (CsA resistant phenotype) by treating target cells with CsA [11,16,17,49]. Unlike the responses observed in our study, however, viral replication of A92E or G94D mutants was generally better in cells treated with high-dose CsA than in untreated target cells.

One factor that has been suggested to modulate the effects of CypA inhibitors in different target cells is the level of expression of CypA [11,21,48,49]. According to this idea, a given virus would replicate optimally in the presence of a limited range of intracellular CypA levels. Thus, in target cells expressing "excessive" amounts of CypA, the treatment of target cells with increasing concentrations of CypA inhibitors might result in an initial improvement in infectivity, followed by a subsequent impairment. Indeed, Ylinen et al. recently demonstrated that 2–3-fold increases in CypA expression in TE671 cells can have a profound impact on the replication of viruses carrying the A92E and G94D Gag mutations [50]. Several findings in our study, however, argued forcefully against the idea that differences in CypA expression, alone, could account for the presence of biphasic dose-response curves. For a given virus, biphasic dose-response curves could be present or absent in target cells expressing similar levels of CypA. For example, CypA expression in U373-X4 and P4 cells was not significantly different at either the mRNA or protein level, but treatment of U373-X4 cells with 8 nM Debio-025 led to a greater than 50% increase in infectivity for 4/8 viruses tested, whereas increases of this magnitude were never seen in P4 cells. In this context, it is noteworthy that the techniques used in this study to quantify Western blots could easily distinguish two-fold differences in CypA levels, indicating that CypA expression in U373-X4 cells and P4 cells was indeed very similar. Similarly, following pretreatment with 8 nM Debio-025, significant increases in infectivity were observed for some viruses in H9 and/or CEM cells, but not P4 cells, despite the fact that CypA expression was significantly lower in these cell types than in P4 cells.

Differences in CypA expression also did not appear to explain the extent that treatment of target cells with high-dose CypA inhibitors inhibited viral infectivity. Treatment of target cells with 200 nM Debio-025 appeared to completely inhibit CypA-CA interactions, because further decreases in viral infectivity were not observed in cells treated with concentrations in the 200 nM – 5 μM range. Nevertheless, for a given virus, the extent of inhibition of viral infectivity could be different in cell types expressing similar amounts of CypA (e.g., the responses of NL4-3 and NRC10 to Debio-025 in U373-X4 cells and P4 cells), and inhibition could be similar in cell types expressing different amounts of CypA (e.g., the responses of NRC3-1 and NRC3-5 to Debio-025 in MT4-R5 cells and P4 cells). Taken together, these findings indicate that differences in CypA expression levels, alone, cannot explain the differences in the dose-response curves observed in our study, but do not exclude a role for CypA levels in modulating these responses.

Our results are consistent with previous studies showing that the modulation of the activity of the restriction factor TRIM5α does not explain the inhibition of viral infectivity in cells treated with high-dose CypA inhibitors. We found that the greatest inhibition of viral replication by target cells treated with high-dose CypA inhibitors occurred in MT4-R5 cells, the cell type expressing the lowest levels of TRIM5α at the mRNA level. Others have shown that human TRIM5α has only modest activity against HIV-1 even after over-expression [7,29,34-36,56]; depleting TRIM5α by RNAi has little effect on HIV replication, including mutants expressing a "CsA-dependent" phenotype, and TRIM5α knockdown does not relieve the negative effect of high-dose CypA inhibitors on viral infectivity [11-13,39,57].

Our results also do not offer support for the possibility that the increase in infectivity of several viruses observed in target cells treated with low-dose Debio-025 resulted from inhibition of the activity of TRIM5α, because TRIM5α mRNA expression was quite similar for cell types in which infection with a given virus did or did not display biphasic dose response curves in response to CypA inhibitors. CypA binding to the HIV-1 CA increases the anti-viral activity of rhesus TRIM5α [8,13,27,57,58], and evidence supporting the idea that CypA binding also increases interactions between CA and human TRIM5α and certain human TRIM5α variants has been presented [27,35,57]. This phenotype has not been observed in all studies, and following overexpression in feline (CRFK) cells, human TRIM5α has been found to have greater inhibitory activity against HIV-1 carrying the G89V mutation, a virus that does not bind CypA, than against wild-type virus [39,57].

It should be emphasized, however, that the extent that low-dose Debio-025 increased infectivity and the residual infectivity observed in cells treated with high-dose Debio-025 were positively correlated. Furthermore, doses of Debio-025 that resulted in an increase in viral infectivity in certain cell types (e.g., U373-X4 cells) overlapped with those that led to an inhibition of infectivity in other target cells (e.g., MT4-R5 cells). Taken together, these findings suggest that the effect of a given dose of Debio-025 on viral replication may reflect the relative abundance of two different putative factors, both of whose effects on viral infectivity can be modulated by blocking CypA-CA interactions, but in opposite directions. Under these conditions, studies seeking correlations between the expression of a single factor (e.g., TRIM5α) and responses to a given dose of a CypA inhibitor may be difficult to interpret, because differences in the level of expression of the second putative factor would also influence the observed response. Others have suggested that CypA-CA interactions could directly influence a variety of processes, including roles in maintaining capsid stability, modulating viral uncoating prior to integration, and protecting viral cores from cellular restriction factors [8,19-23]. Thus, activities directly attributable to CypA may represent one of these factors. Further studies are needed to identify the additional putative cellular activity (or activities) involved in the biphasic responses observed here.

## Conclusion

In conclusion, our studies indicate that interactions between host-cell factors and viral proteins coded by *gag *have an important effect on viral infectivity, and the studies evaluating the effect of CypA inhibitors suggest that at least two distinct cellular activities interacting with the CA protein may be involved. Thus, the impact on viral infectivity resulting from treating a given cell type with CypA inhibitors appears to be multifactorial. This reflects both the relative abundance of two distinct activities, whose effect on viral infectivity is influenced in opposite directions by CypA-CA interactions, as well as the intrinsic sensitivity of a given virus to each of these cellular activities.

## Methods

### Cell culture

HeLa cells, HeLa-derived P4 cells, 293T cells and U373-X4 cells were cultured in Dulbecco's modified Eagle's medium supplemented with 10% fetal calf serum, 100 U/ml penicillin G and 100 μg/ml streptomycin (complete medium). MT4-R5 cells, H9 cells and CEM cells were cultured in similarly supplemented RPMI-1640 medium. For U373-X4 cells stably expressing CXCR4, the medium also contained 10 μg/ml puromycin and 100 μg/ml hygromycin B. For P4 cells, the medium also contained 500 μg/ml G418.

### Production of recombinant viruses

The vector pNL4-3-ΔENV-lucR, which expresses *Renilla *luciferase in place of Nef and carries a large deletion in env, has previously been described [59]. To restore the NL4-3 envelope, the *EcoR*I – *BamH*I fragment from pNL4-3 was ligated into similarly cleaved pNL4-3-ΔENV-lucR, creating pNL4-3-lucR. To facilitate the construction of Gag-PR recombinants, a *Cla*I site was introduced by site-directed mutagenesis immediately downstream of the protease region of pNL4-3-lucR as previously described [60], creating pNL4-3-lucR-XC and pNL4-3-ΔENV-lucR-XC.

To generate recombinant viruses expressing Gag-PR sequences from different patients, a protocol analogous to that described by Zennou et al [60] was used. Briefly, plasma was obtained from 5 patients followed at Hôpital Bichat-Claude Bernard, Paris, France who met the following criteria: i) infected with a subtype B virus; ii) naïve to antiretroviral therapy; iii) absence of resistance mutations in the protease. RNA was purified from plasma, and the HIV-1 sequence encompassing Gag and protease was amplified by RT-PCR using the primers ProC and BssHII. The PCR products were digested with *BssH*II and *Cla*I, and the fragment used to replace the corresponding region from both pNL4-3-lucR-XC (viruses expressing the NL4-3 envelope) and pNL4-3-ΔENV-lucR-XC (viruses in which the NL4-3 envelope is deleted). For one patient, two different recombinant viruses were evaluated (NRC3-1 and NRC3-5). A recombinant virus carrying the pNL4-3 Gag-PR sequence in which 3 protease resistance mutations (L10I, G48V and V82A) had been introduced by site-directed mutagenesis (GPCA) was constructed in a similar fashion. The resulting plasmids obtained using the pNL4-3-lucR-XC backbone were used to transfect 293T cells. To produce VSV pseudotyped env-deleted viruses, the constructs obtained using the pNL4-3-ΔENV-lucR-XC backbone were cotransfected with a VSV-G expression plasmid (phCMV-G). Virus-containing culture supernatants were harvested 48 h after transfection, passed through 0.45 μm pore size filters, adjusted to 500 ng p24/ml and stored at -80°C until use.

### Infectivity assay

The infectivity of recombinant viruses for different target cell types was determined by measuring luciferase activity. P4 cells (9 × 10^3^/well), U373-X4 cells (4.5 × 10^3^/well) or MT4-R5 cells (2.5 × 10^4^/well) were seeded in black-wall, clear bottom 96-well plates (Greiner, Frickenhausen, Germany) 24 hours prior to infection. Cells were infected with serial two-fold dilutions of each virus stock (final concentrations: 1.56 – 100 ng p24/ml for viruses expressing the NL4-3 envelope; 0.156 – 10 ng p24/ml for VSV pseudotyped viruses) in a final volume of 200 μl culture medium and incubated at 37°C. After 40 hours, the supernatant of adherent cells was completely removed and 50 μl of 1× lysis buffer (Renilla Luciferase kit, (Promega, Madison, WI) were added. MT4-R5 cells were centrifuged (1200 × g; 5 min), 150 μl of culture medium were removed, and 50 μl of 2× lysis buffer were added to each well. Plates were maintained at room temperature for 30 min, after which wells were sequentially injected with 100 μl of luciferase substrate (Promega), and 3 seconds later, light emission (relative light units, RLU) was measured over a two sec interval using a Microlumat LB96P luminometer (Berthold, Oak Ridge, TN). Each sample was evaluated in triplicate. RLU were plotted as a function of amount of p24 used to infect the cells, and infectivity was defined as the slope [(RLU/2 sec)/(ng p24/ml)] as determined by linear regression; in this analysis, each replicate RLU value was treated as an individual point.

To facilitate the comparison of results obtained in different experiments, the infectivity of pNL4-3 was determined on each 96-well plate, and the infectivity value obtained for this standard was used to normalize the infectivity of the other viruses evaluated on the same plate. To do so, the average infectivity of pNL4-3 for each cell type was determined (4 independent experiments; 3 96-well plates/experiment; n = 12), and used to calculate a correction factor for each plate as follows: [(infectivity for NL4-3 on the given 96-well plate)/(average infectivity for NL4-3)]. The infectivity of the other viruses evaluated on the same plate multiplied by this correction factor is referred to as normalized infectivity.

### Effect of DEBIO-025 and cyclosporine A on viral Infectivity

Debio-025 was kindly provided by Debiopharma (Lausanne, Suisse). Cyclosporine A was obtained from Sigma-Aldrich (St. Louis, MO). Both were dissolved in DMSO (final concentration 10 mM), and stored in aliquots at -80°C. P4 cells (9 × 10^3^/well), U373-X4 cells (4.5 × 10^3^/well), H9 cells (5 × 10^4^/well), MT4-R5 cells and CEM cells (2.5 × 10^4^/well) were seeded in 96-well plates 24 h prior to infection. Cells were pre-treated with serial dilutions of Debio-025 or CsA for 15 min and infected with 100 ng p24/ml (patient-derived recombinant viruses) or 20 ng p24/ml (NL4-3) of virus in the presence of the same dilutions of Debio-025 or CsA. After 40 hours of incubation at 37°C, culture supernatants were removed, the cells were lysed, and luciferase activity expressed by the cell lysates was evaluated as described above. Results are expressed as the percentage of luciferase activity observed in non-pretreated cells. For studies evaluating the effects of CsA, cells were treated with serial four-fold dilutions (final concentrations 8 nM – 2 μM). For studies evaluating Debio-025, several dilution schemes were used in the course of these studies. For the presentation of the data and statistical analyses, results obtained for the indicated ranges of concentrations of Debio-025 were pooled, and are labelled as follows: 5μM (2–5 μM), 1μM (0.5–1 μM); 200 nM (125–200 nM); 40 nM (30–40 nM); 8 nM (8–10 nM); 1.6 nM (1.6 nM). All experiments were conducted in triplicate and repeated 6 times (U373-X4, P4, MT4-R5) or three times (CEM, H9). Neither Debio-025 nor CsA displayed cytotoxic activity against the target cells over the range of concentrations used, as evaluated using a MTT assay (Sigma).

### Quantification of CypA and TRIM5α mRNA in target cells

Cell suspensions were washed with PBS and mRNA was extracted from 2 × 10^6 ^cells using NucleoSpin RNA II kits (Macherey-Nagel, Düren, Germany). cDNA synthesis was performed using random hexamers and Moloney murine leukemia virus reverse transcriptase (Invitrogen, Carlsbad, CA), according to the manufacturer's instructions. CypA, TRIM5α and GAPDH cDNAs were quantified by real-time PCR using the primers and Taqman probes shown in Table 2. DNA was diluted 1:10 in 10 mM Tris-HCl (pH 8.0) containing 0.1 mM EDTA and 100 ng/ml salmon sperm DNA. Reaction mixtures (final volume, 50 μl) contained 1× ABsolute QPCR ROX mix (Abgene, Thermo-Fisher, Rockford, IL.), 200 nM (each) primer, 100 nM Taqman probe, and 10 μl of diluted DNA. Amplification was performed with a 7000 sequence detection system (Applied Biosystems). Cycling conditions were as follows: 50°C for 2 minutes, 95°C for 10 minutes, and 40 cycles at 95°C for 15 seconds and 60°C for 1 minute each. To serve as standards, cDNAs for CypA and TRIM5α were cloned into pCR-TOPO2.1 vectors (Invitrogen), and a GAPDH cDNA sequence encompassing that recognized by the GAPDH primers was inserted into the polylinker. Thus, the same serial dilutions of linearized plasmids could be used as standards for the quantification of GAPDH and the gene of interest. Results are expressed as the number of copies of CypA or TRIM5α mRNA per 1000 copies of GAPDH mRNA.

**Table 2:** Primers and probes used to quantify viral sequences by real-time PCR

**GAPDH**	
forward primer	5'- ACCCCTGGCCAAGGTCATC
reverse primer	5'- AGGGGCCATCCACAGTCTTC
Probe	5'- (6-Fam)AGGACTCATGACCACAGTCCATGCCA(Tamra)
**CypA**	
forward primer	5'- GGCCGCGTCTCCTTTGA
reverse primer	5'- AATCCTTTCTCTCCAGTGCTCAGA
Probe	5'- (6-Fam)TGCAGACAAGGTCCCAAAGACAGCAG(Tamra)

**TRIM5α**	
forward primer	5'- TGCCTCTGACACTGACTAAGAAGATG
reverse primer	5'- GGGCTAAGGACTCATTCATTGG
Probe	5'- (6-Fam)AAGCTTTTCAACAGCCTTTCTATATCATCGTGTGATA(Tamra)

### Western blotting

Cell suspensions were washed two times with PBS, and 3 × 10^6 ^cells were pelleted and resuspended in 150 μl of lysis buffer (1% NP40, 150 mM NaCl, 50 mM of Tris-HCl – pH 7.8). Lysates were cleared by centrifugation (9300 × g; 10 min), and protein content was determined using the Bradford assay (Bio-Rad, Hercules, CA). Cellular proteins were separated by electrophoresis into 10% SDS-PAGE gels, and transferred to Immobilion-P membranes (Millipore, Bedford, BA). The blots were probed sequentially with a rabbit anti-cyclophilin A antibody (1:1000 dilution, Cell Signaling Technology, Danvers, MA) and a IRDye 800CW-conjugated donkey anti-rabbit IgG antibody (1:15000 dilution, Li-Cor Biosciences, Lincoln, NE). The blots were scanned using an Odyssey Infrared imaging system, and fluorescence intensity was analysed using Odyssey application software (version 2.1, Li-Cor). Lysates from P4 cells were included in each gel, and the intensities of the CypA bands in the other samples on the same gel are expressed relative to that observed for P4 cells. The results presented are the mean of 4 independent experiments, each performed using different cell suspensions.

### Statistical analysis

Results are expressed as mean ± SD unless otherwise indicated. Comparisons between groups were performed by ANOVA. Post test comparisons, performed only if p < 0.05, were made using Bonferroni's Multiple Comparison Test. Correlations were evaluated using the Pearson test.

## Competing interests

The authors declare that they have no competing interests.

## Authors' contributions

SM and ED constructed the recombinant viruses and performed the infectivity studies and helped write the manuscript. DL performed the real-time PCR studies. FC and AH participated in its design and supervision of the studies and wrote the manuscript. All authors read and approved the final manuscript.
